# Acute Stage Longitudinal Change of Quality of Life from Pre- to 3 Months after Surgical Treatment in Head and Neck Cancer Patients

**DOI:** 10.31557/APJCP.2019.20.10.3129

**Published:** 2019

**Authors:** Yuichi Tashimo, Yoshiaki Ihara, Ken Yuasa, Shinji Shinji, Yoshiro Saito, Hideyuki Katsuta, Toshikazu Shimane, Koji Takahashi

**Affiliations:** 1 *Division of Oral Rehabilitation Medicine, Department of Special Needs Dentistry, Showa University School of Dentistry, *; 2 *Head and Neck Oncology Center, Showa University Hospital, Tokyo, Japan. *

**Keywords:** Quality of life, muscle mass, oral function, feeding function, head and neck cancer

## Abstract

**Purpose:**

Head and neck cancer (HNC) patients experience various posttreatment side effects that decrease quality of life (QOL). Some previous study reported that QOL of HHC patients were returned baseline (before treatment) after a year post treatment. However, acute stage longitudinal changes of QOL in HNC patients remains unclear. This point might be important for early reintegration of HNC patients. This study aimed to investigate the acute stage longitudinal change of the relationship between QOL and oral function in HNC patients had surgery.

**Methods:**

45 HNC patients (23 men) scheduled for surgical treatment were enrolled in this study. Primary tumor sites were 22 tongue, 5 maxilla, 4 mandible, 3 pharynx and others. Weight, body mass index (BMI), whole body soft lean mass (SLM), and skeletal muscle mass (SMM) were evaluated as muscle mass-related measurements. Lip closure force (LC) and tongue pressure (TP) were evaluated as oral function measurements. Feeding function was evaluated using the Functional Oral Intake Scale (FOIS). QOL was assessed using the European Organization for Research and Treatment of Cancer QOL Questionnaire QLQ-C30 and H&N 35. Measures were evaluated at pre-surgical treatment (PT), and 1 month (1M) and 3 months (3M) after surgery. The change of QOL parameters and relationships between measurements were assessed.

**Results:**

For QOL assessments, role functioning, fatigue, speech problems, trouble with social eating, trouble with social contact, and opening mouth significantly decreased from PT to 1M, but significantly increased from 1M to 3M. Weight, BMI, SLM, SMM, LC, TP, and FOIS demonstrated significant relationships with QOL from PT to 1M. Meanwhile, from 1M to 3M, weight, BMI, SLM, SMM, LC, and FOIS showed significant relationships with QOL assessments.

**Conclusions:**

Both oral function and muscle mass-related measurements significantly affected QOL in HNC patients.

## Introduction

Currently, both the prevalence and survival rates of head and neck cancer (HNC) are increasing (Young et al., 2015) owing to advances in medical technology (American Cancer Society. Cancer fact & Figure, 2016; Van den Berg et al., 2014). HNC markedly affects not only oral function, but also the cosmetic and psychological aspects (Rütten et al., 2011; Crary et al., 2014). The acute side effects of treatment may persist beyond treatment, while additional chronic effects may develop after at least 90 days after treatment discontinuation (Ganzer et al, 2015; Glastonbury et al., 2010). Common oral morbidities resulting from HNC treatment include oral pain, oral dryness, and altered taste and smell perception. One of the most prevalent and debilitating side effects of HNC treatment is dysphagia (i.e., swallowing difficulty) (Ihara et al., 2018) that may develop as both acute and chronic complication of HNC treatment (Hutcheson et al.,2012; Van den Berg et al., 2014). Dysphagia has been reported in over 76% of HNC patients treated with concurrent chemotherapy (CRT). It decreases the patient’s quality of life (QOL) following HNC treatment (Kalyanam et al., 2018; Anuradha et al., 2013). QOL is considered to be an important factor in both treatment decision and outcome evaluation (Anuradha et al., 2013; Blazeby et al., 1995; Maciejewski et al., 2010; Gonçalves et al., 2012). Particularly, QOL is necessary in multidirectional analysis and appropriate evaluation of treatment results.

The result of HNC treatment should be evaluated according to both QOL and posttreatment functional outcomes (Guenzel et al., 2018). However, only few studies have conducted a multidirectional analysis that include QOL before and after HNC treatment. Further, majority of previous studies focused on HNC patients who received chemoradiation therapy (Van den Berg et al., 2014; Rütten et al., 2011; Ihara et al., 2018; Hutcheson et al., 2012; Kalyanam et al., 2018). Some previous study reported that QOL of HNC patients were returned baseline (before treatment) after a year post treatment (Gritz et al., 1999; Martine et al., 2019; Marzouki et al., 2018). However, the acute stage association between QOL and other functions in HNC patients who underwent surgery remains unclear. This point might be important for early reintegration of HNC patients.

This study aimed to investigate the longitudinal change of QOL for acute stage in HNC patients who underwent surgery by conducting a multidirectional analysis of pre- and posttreatment QOL. 

## Materials and Methods

*Patients*

This study included HNC patients who were scheduled for surgical treatment at the Head and Neck Oncology Center, Showa University Hospital and were then referred to the Department of Special Needs Dentistry, Division of Oral Rehabilitation Medicine, Showa University Dental Hospital for rehabilitation. The exclusion criteria were (1) age < 20 years, (2) inability to follow instructions, (3) other malignant tumors, (4) severe systemic diseases that may influence the evaluation, and (5) incomplete measurement data. 

*Assessments*

All measurements were performed by dentists of the Department of Special Needs Dentistry, Division of Oral Rehabilitation Medicine, Showa University Dental Hospital. The primary tumor site, TNM Classification, method of surgical operation, and medical history were collected from the medical records. The patient’s weight, body mass index (BMI), whole body soft lean mass (SLM), and skeletal muscle mass (SMM) were evaluated as muscle mass-related measurements. Lip closure force (LC) and tongue pressure (TP) were evaluated as oral function measurements. Feeding function was evaluated using the Functional Oral Intake Scale (FOIS), while QOL was assessed using the European Organization for Research and Treatment of Cancer (EORTC) QOL Questionnaire QLQ-C30 and QLQ-H&N 35.

Patients were examined at pre-surgical treatment (PT; 2 weeks to 2 days before surgery), a month after surgery (1M), and 3 months after surgery (3M). 

*Muscle mass-related measurements*

SLM and SMM were measured using Inbody S20 (BioSpace, Seoul, Korea), which can evaluate the patient’s SLM and SMM in supine position. The patients were placed in the supine position on the examination table, with four electrodes on the first and third fingers and four points on the left and right ankles, totaling to 8 contact-type electrodes (Okamoto et al., 2006). The patient’s weight and BMI were measured at each time point. Changes in body weight and percentage of body weight from PT to each time point were calculated.

*Oral function measurements*

LC was measured 5 times using a lip force measuring device (Lip de Cum model LDC-110R, Cosmo-Instruments Co, Ltd, Tokyo, Japan). The average score of the 5 measurements was then calculated as the LC score (Morisaki et al., 2015; Ono et al., 2003).

TP was evaluated using the JMS tongue pressure measuring device (JMS Co. Ltd., Hiroshima, Japan). The balloon-shaped intraoral probe was placed behind the upper front teeth. Patients were instructed to push the probe with the maximum force between the hard palate and tongue, and changes in air pressure inside the probe was measured. The measurement was performed 10 times, and the average score was calculated as the TP score (Hasegawa et al., 2017).

*Feeding function*

The FOIS was used as a measure of functional eating status (Crary et al., 2005). The FOIS is a valid and reliable tool used to document functional eating abilities. A 7-point ordinal scale describes the functional oral intake of patients with dysphagia.

*QOL measurements*

QOL was assessed using the Japanese version of EORTC QLQ-C30 version 3.0 and QLQ-H&N35 questionnaires. The scores were calculated according to the EORTC scoring manual (Aaronson et al., 1993; Fayers et al., 2001).

*Statistical analysisqa*

Univariate analyses of potential associations were conducted using t-tests for the comparison of all measurements at each time point. Spearman’s rank correlation coefficient was used to evaluate the relationships among QOL measurements that significantly decreased after HNC treatment and other measurements from PT to 1M and 1M to 3M. Statistical analyses were performed using IBM SPSS version 25 (IBM, New York, USA). All p values were two-sided, and p < 0.05 was considered significant.

## Results

*Patients*

A total of 45 patients (23 men and 22 women) were included in the study. The mean patient age was 66.51 years (SD: 12.5 years). The primary tumor site was the tongue, maxilla, mandible, pharynx, and others in 22, 5, 4, 3, and 11 patients, respectively. The patients’ characteristics are detailed in Table 1. 

*Muscle mass-related measurements*

**Figure 1:**
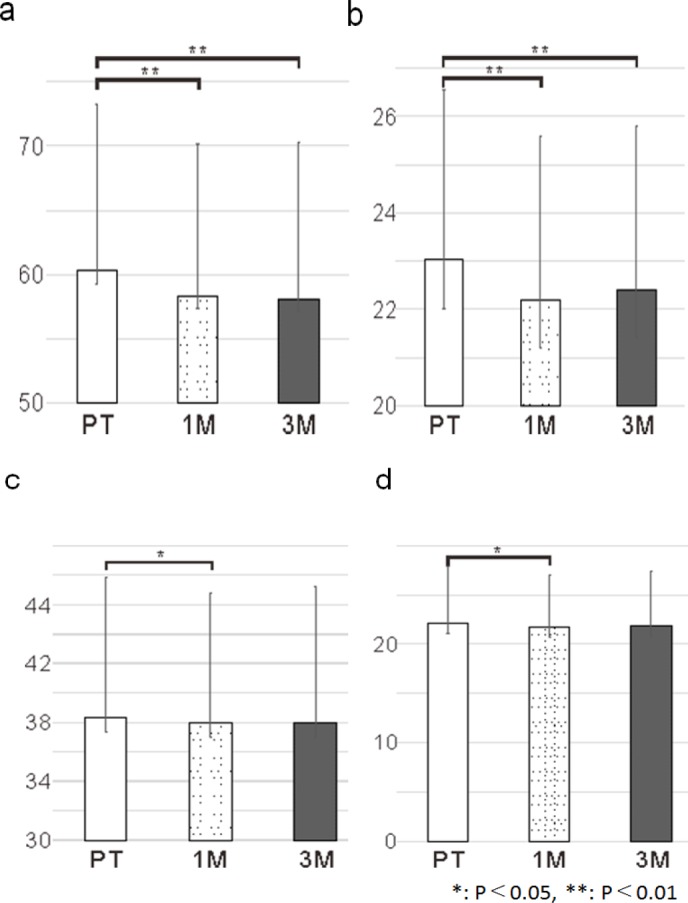
Muscle Mass Related Measurement Outcomes. a, Change in weight; b, Change in BMI; c, Change in SLM; d, Change in SMM

Weight: At PT, the average weight was 60.27 kg (SD = 13.02 kg). A significant reduction was observed at 1M (mean: 58.32 kg, SD = 11.82 kg; t = 5.41, p <0 .001), while no significant increase was noted from 1M to 3M (mean: 58.10 kg, SD = 12.23 kg; t = -0.59, p = 0.560). In addition, the average weight was significantly reduced from PT to 3M (t = 3.86, p < 0.001; Figure 1a).

BMI: At PT, the average BMI was 23.02 kg/m^2^ (SD = 3.52 kg/m^2^). A significant reduction was observed at 1M (mean: 22.20 kg/m^2^, SD = 3.41 kg/m^2^; t=5.75, p < 0.001). However, the average BMI was not significantly increased at 3M (mean: 22.40 kg/m^2^, SD = 3.40 kg/m^2^) than that at 1M (t = 0.29, p = 0.770). Furthermore, the average BMI at 3M was significantly reduced from that at PT (t = 3.95, p < 0.001; Figure 1b).

SLM: At PT, the average SLM was 38.32 kg (SD = 9.52 kg). A significant reduction was observed at 1M (mean: 38.00 kg, SD = 8.81 kg; t = 2.10, p = 0.040)). Meanwhile, no significant change in the average SLM was observed at 3M compared to that at 1M (mean: 38.00 kg, SD = 9.22 kg; t = 1.57, p = 0.125). Furthermore, the average SLM at 3M showed no significant changes (t = 1.30, p = 0.200) compared to that at PT (Figure 1c).

SMM: At PT, the average SMM was 22.10 kg (SD = 5.82 kg). A significant reduction was observed at 1M (mean: 21.72 kg, SD = 5.23 kg; t = 2.59, p = 0.010). Meanwhile, there was no significant reduction in the average SMM at 3M (mean: 21.79 kg, SD = 5.57 kg; t = -1.97, p = 0.056) compared to that at 1M. Furthermore, no significant change in the average SMM at 3M compared to that at PT was noted (t = 1.66, p =0 .100; Figure 1d).

*Oral function measurements*

**Figure 2:**
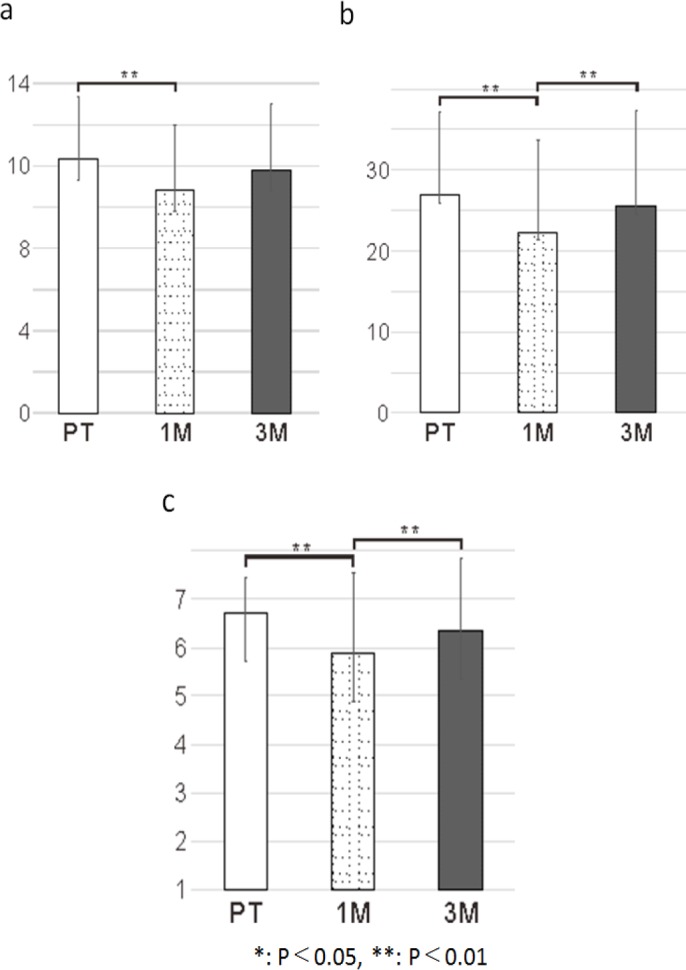
Oral Function Measurements and Feeding Outcomes. a, Change in LC; b, Change in TP; c, Change in FOIS

LC: At PT, the average LC was 12.33 N (SD = 3.03 N). A significant reduction was observed at 1M (mean: 10.80 N, SD = 3.19 N; t = 3.47, p =0 .001). Meanwhile, the average LC showed no significant change at 3M compared to that at 1M (mean: 11.79 N, SD = 3.27 N; t = -1.73, p =0.092). Furthermore, the average LC at 3M showed no significant change compared to that at PT (t = 1.56, p =0 .127; Figure 2a).

TP: At PT, the average TP was 26.89 kPa (SD = 10.21 kPa). A significant reduction (was observed at 1M (mean: 22.30 kPa, SD = 11.43; t = 4.23, p < 0.001). At 3M, the average TP was significantly increased (mean: 25.47 kPa, SD = 12.23; t = -3.17, p = 0.003) compared to that at 1M. Meanwhile, the average TP at 3M showed no significant change compared to that at PT (t = 1.65, p = 0.107; Figure 2b).

*Feeding function*

FOIS: At PT, the average FOIS was 6.73 (SD = 0.72). At 1M, the mean FOIS was significantly decreased (mean: 5.89; SD = 1.49; t = 4.07, p < 0.001). Meanwhile, the mean FOIS score at 3M was significantly increased compared to that at 1M (mean: 6.36, SD = 1.49; t = -3.17, p = 0.003). Furthermore, the average FOIS at 3M was not significantly different from that at PT (t = 1.88, p = 0.068; Figure 2c).

*QOL measurements*

**Figure 3:**
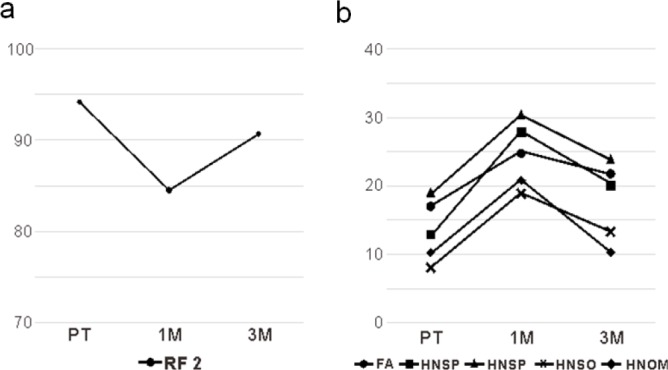
QOL Measurement Outcomes. a, Significant change in functional scale both PT to 1M and 1M to 3M; b, Significant change in symptom scales both PT to 1M and 1M to 3M

Table 2 presents the results of EORTC QLQ-C30. Table 3 presents the results of EORTC QLQ-H&N35. For QOL measurements, no significant change was noted from PT to 1M in Global health status (p = 0.768). However, it increased significantly from 1M to 3M (p = 0.039). For functional scales, physical functioning (PF 2) and role functioning (RF 2) decreased significantly from PT to 1M (PF 2; p = 0.001, RF 2; p = 0.004), while RF2, emotional functioning (EF), and social functioning (SF) increased significantly from 1M to 3M (RF 2; p = 0.005, EF; p = 0.048, SF; p = 0.007). Only RF2 indicated a significant change in both PT to 1M and 1M to 3M (Figure 3a). In symptom scales, fatigue (FA), dyspnea (DY), senses problems (HNSE), speech problems (HNSP), trouble with social eating (HNSO), trouble with social contact (HNSC), opening mouth (HNOM), and weight gain (HNWG) decreased significantly from PT to 1M (FA; p = 0.004, DY; p = 0.011, HNSE; p = 0.021, HNSP; p < 0.001, HNSO; p = 0.027, HNSC; p = 0.001, HNOM; p = 0.009, HNWG; p = 0.010). Furthermore, FA, pain (PA), insomnia (SL), appetite loss (AP), swallowing (HNSW), HNSP, HNSO, HNSC, and HNOM increased significantly from 1M to 3M (FA; p = 0.011, PA; p = 0.022, SL; p = 0.037, AP; p = 0.027, HNSW; p = 0.010, HNSP; p = 0.001, HNSO; p = 0.043, HNSC; p < 0.001, HNOM; p = 0.002). In addition, FA, HNSP, HNSO, HNSC, and HNOM showed significant change in both PT to 1M and 1M to 3M (Figure 3b).

**Table 1:** Patient Characteristics (N=45 )

Variables	N=45
Gender (Male : Female)	(23: 22)
Age (mean, SD, range )	66.51, 12.50, 36-85
Tumour site	22
Tongue	5
Maxilla	4
Mandible	3
Pharynx	6
Thyroid	2
Oropharynx	2
hypopharynx	1
Salivary gland	
Tumour size	2
T	15
is	13
1	7
2	5
3	3
4a	34
4b	11
N	
0	
+	
Tumour stage	2
0	12
I	12
II	9
III	8
IVA	2
IVB	

**Table 2:** The result of EORTC QLQ-C30. indicated Global Health Status. indicated functional scales. Indicated symptom scales

QOL items	Scale name	PT	1M	3M
		Mean (SD)	Mean (SD)	Mean (SD)
Global health status	QL2	66.08 (23.32)	68.41 (22.77)	73.61 (19.91)
Physical functioning	PF2	96.12 (6.78)	91.47 (9.46)	90.32 (12.95)
Role functioning	RF2	94.19 (18.12)	84.50 (19.57)	90.65 (17.43)
Emotional functioning	EF	80.81 (21.23)	86.91 (17.57)	89.09 (13.07)
Cognitive functioning	CF	87.60 (17.46)	87.21 (17.84)	87.30 (14.62)
Social functioning	SF	86.82 (21.89)	86.82 (17.37)	90.87 (16.10)
Fatigue	FA	17.05 (14.79)	25.06 (18.98)	21.69 (19.84)
Nausea and vomiting	NV	2.33 (7.78)	2.71 (15.39)	3.17 (9.18)
Pain	PA	13.18 (20.51)	19.77 (18.38)	13.89 (18.11)
Dyspnoea	DY	5.43 (16.13)	12.40 (21.76)	10.32 (21.39)
Insomnia	SL	13.18 (17.90)	16.26 (24.72)	14.17 (23.62)
Appetite loss	AP	11.11 (17.44)	15.50 (20.88)	8.73 (16.50)
Constipation	CO	12.40 (20.51)	14.73 (22.07)	12.70 (25.37)
Diarrhoea	DI	4.65 (11.66)	6.20 (14.97)	7.142 (13.80)
Financial difficulties	FI	18.60 (30.98)	8.53 (20.33)	9.52 (24.69)

**Table 3:** The Result of EORTC QLQ-H&N35

QOL items	Scale name	PT	1M	3M
		Mean (SD)	Mean (SD)	Mean (SD)
Pain	HNPA	13.95 (16.70)	13.18 (15.22)	9.32 (11.76)
Swallowing	HNSW	14.15 (21.37)	19.44 (25.42)	14.55 (21.19)
Senses problems	HNSE	4.26 (9.66)	11.11 (17.82)	8.73 (13.33)
Speech problems	HNSP	12.66 (23.99)	28.03 (26.18)	20.37 (20.51)
Trouble with social eating	HNSO	18.80 (20.64)	30.36 (22.39)	23.81 (18.70)
Trouble with social contact	HNSC	8.06 (15.86)	18.97 (23.46)	13.33 (21.59)
Less sexuality	HNSX	20.15 (29.95)	17.86 (25.72)	21.83 (28.81)
Teeth	HNTE	7.75 (18.98)	11.63 (21.60)	8.73 (16.10)
Opening mouth	HNOM	10.08 (22.41)	20.93 (24.28)	10.32 (21.14)
Dry mouth	HNDR	20.15 (26.37)	26.36 (31.06)	26.98 (29.65)
Sticky saliva	HNSS	22.48 (32.92)	23.25 (29.35)	19.05 (26.52)
Coughing	HNCO	10.08 (17.04)	12.40 (19.18)	11.11 (20.31)
Felt ill	HNFI	15.50 (24.38)	22.48 (25.89)	17.46 (24.55)
Pain killers	HNPK	7.75 (14.20)	8.53 (14.20)	3.17 (9.89)
Nutritional supplements	HNNU	3.88 (10.79)	5.43 (12.42)	3.97 (9.89)
Feeding tube	HNFE	0.78 (5.08)	1.55 (7.10)	1.59 (7.18)
Weight loss	HNWL	8.53 (14.65)	8.53 (14.65)	6.35 (13.21)
Weight gain	HNWG	4.65 (11.66)	11.63 (15.97)	11.11 (15.81)

**Table 4:** Between PT and 1M, the Measurement Items that Positive Correlated with the Significantly Decreased QOL Items between PT and 1 M

QOL items	Measurement items	correlation coefficients	P value
PF2	Weight	0.49	0.001
	BMI	0.485	0.001
	LC	0.369	0.019
	TP	0.581	<.001
	FOIS	0.419	0.007
RF2	TP	0.32	0.044
	FOIS	0.386	0.014
FA	TP	-0.33	0.038
HNSE	BMI	-0.308	0.05
	TP	-0.327	0.039
HNSP	SLM	-0.344	0.03
	SMM	-0.344	0.03
	LC	-0.324	0.042
	TP	-0.424	0.006
	FOIS	-0.366	0.02
HNSO	SLM	-0.382	0.018
	SMM	-0.354	0.029
	FOIS	-0.376	0.02
HNSC	Weight	-0.512	0.001
	BMI	-0.537	<.001
	SLM	-0.385	0.014
	SMM	-0.415	0.008
	LC	-0.365	0.021
	TP	-0.615	<.001
	FOIS	-0.681	<.001
HNOM	Weight	-0.33	0.035
	SLM	-0.369	0.019
	SMM	-0.375	0.017
	TP	-0.342	0.031

**Table 5:** Between 1M and 3M, the Measurement Items that Positive Correlated with the Significantly Increased QOL Items

QOL items	Measurement items	correlation coefficients	P value
RF2	Weight	-0.517	0.001
	BMI	-0.436	5
EF	FOIS	0.45	4
SF	FOIS	0.445	0.005
FA	FOIS	-0.339	0.035
PA	FOIS	-0.34	0.034
SL	LC	-0.328	0.048
	FOIS	-0.549	0.001
AP	FOIS	-0.427	0.007
HNSW	FOIS	-0.351	0.033
HNSP	SLM	-0.323	0.039
HNSO	SLM	-0.342	0.031
	SMM	-0.332	0.036
HNSC	FOIS	-0.341	0.034

*Correlation between QOL items and other measurement items*

From PT to 1M, PF2 showed significant strong correlation with weight (r = 0.490, p = 0.001), BMI (r = 0.485, p = 0.001), TP (r = 0.581, p < 0.001), and FOIS (r = 0.419, p = 0.007). HNSP showed significant strong correlation with TP (r = - 0.424, p = 0.006). HNSC also showed significant strong correlation with weight (r = - 0.512, p = 0.001), BMI (r = - 0.537, p < 0.001), SMM (r = - 0.415, p = 0.008), TP (r = - 0.615, p < 0.001), and FOIS (r = - 0.681, p < 0.001) (Table 4). From 1M to 3M, RF2 demonstrated significant correlation with weight (r = - 0.497, p = 0.001) and BMI (r = - 0.447, p = 0.004). Further, EF, SF, SL, and AP demonstrated significant correlation with FOIS (EF: r = 0.552, p < .001; SF: r = 0.517, p = 0.001; SL: r = - 0.549, p = 0.001; and AP: r = - 0.427, p = 0.007) (Table 5). 

## Discussion

The reduction of oral function might be related to the surgical region of HNC. In this study, patients underwent only surgical treatment, and although oral function decreased after surgical treatment, it was recovered after 3 months. Unlike external beam radiation therapy and CRT, surgical treatment has less additional chronic effects on oral function (Luo et al., 2016). However, surgical treatment has strong acute side effects such as wound pain, and this might have influenced the result of this study. Moreover, the patients in this study underwent oral function rehabilitation, such as tongue strength training, lip closure training, and respiratory muscle strength training. These rehabilitations helped to improve oral function. In addition, majority of patients in this study (58%) had stage I or II. These patients treated with free tissue transfer. It was reported that single-stage reconstruction of head and neck like free tissue transfer reconstruction defected with much greater success and less morbidity (Chim et al., 2010).

The QOL of HNC patients has been reported to decrease after treatment and did not recover to baseline level (Loorents et al., 2016). In this study, the same tendency was noted in the symptom scale evaluation items. At 1M, the FA, DY, HNSE, HNSP, HNSO, HNSC, HNOM, and HNWG were significantly decrease from that at PT. One possible reason might be that patients were still not fully recovered at this time point because of anatomical changes in the pharynx and oral cavity, decrease of dexterity, limitations in range of movement, and decrease in moving speed. 

From PT to 1M, significant relationships were noted between QOL assessments and other measurements (14 items of muscle mass related measurements, 10 items of oral function measurements, and 5 items of feeding function). These results indicate that both muscle mass related measurements and oral function measurements had significant effects on QOL, and these functions were not recovered from at 1 month after surgical treatment. In this study, because of pain and/or healing process of wound area, over a week was necessarily to begin rehabilitation after surgical treatment for patients who underwent minor surgical treatment like partial glossectomy. Moreover, it is thought that a longer time was necessary to begin rehabilitation after surgical treatment for patients who underwent major surgery such as reconstructive surgery of the oral cavity. The common oral morbidities during the early stage of HNC treatment include dysphagia, oral pain, and oral dryness (Luo et al., 2016). This might have caused the significant association between oral function and QOL. Moreover, some patients were still hospitalized at 1 month postoperative, and others were placed on tube feeding, causing difficulty in achieving adequate nutrition. In addition, some patients needed modified diets, which might have caused the significant association of BMI and weight with QOL. 

Meanwhile, from 1M to 3M, different relationships were noted between QOL assessments and other measurements (5 items of muscle mass-related measurements, one item of oral function measurements, and 8 items of feeding function). Particularly, the evaluation measurements of oral function decreased to only a measurement (LC) between 1M and 3M. This indicated that a decrease in oral function had significant effect on QOL at the early stage following treatment. However, the effect became weak at 3 months postoperative. One possible reason might be that rehabilitation of oral function improves oral function (TP, LC). Nevertheless, feeding function (FOIS), which involves complex movement (both oral and pharyngeal), remained significantly correlated with QOL, indicating that oral function requiring complex movements such as feeding, speech, and social contact had stronger effect on QOL than simple function such as TP and LC. It was reported that social oral function, such as speech and eating, had strong effects on QOL during the late stage after treatment (Carnaby and Crary, 2014). Similar results were obtained in this study.

The difference in correlation between PT - 1M and 1M - 3M is considered to be primarily due to functional deterioration because of surgical treatment and changes in the social environment. A previous study suggested that factors influencing QOL assessments were highly correlated with the time period after surgery and social environment of patients after social reversion (List et al., 2000). In this study, single function such as LC and TP showed a significant correlation with change in QOL item at 1M. However, only LC indicated significant correlation to QOL assessment. Meanwhile, measurement items involving many factors (FOIS, SMM, and SLM) were correlated with QOL. Other QOL items correlated with other measurements did not change at PT - 1M and 1M - 3M. As for correlation coefficients, no factors showing strong correlation were recognized. This point might indicate that the QOL of HNC patients who underwent surgical treatment is influenced by multiple factors, and not a single factor. This means that improving the QOL of HNC patients require a multifactorial approach, and strategies need to be patterned according to the time posttreatment. Dysphagia is among the most prevalent and debilitating symptoms resulting from HNC treatment. It has been reported that different mechanisms may contribute to the development and maintenance of dysphagia during HNC treatment (Ihara et al., 2018). The pattern in correlation between QOL and functional assessment items differed according to the time point after treatment.

The result of this study showed that no significant change was noted from PT to 1M in Global health status though many functional measurements significantly decreased. And, it increased significantly from 1M to 3M. One of the possible of this reason might be that psychological affect had more effect than functional measurement on it. A previous study reported that the surgery of cancer had a physical psychological stress on patients (Kubota et al.,1988). Patients in this study might had a stress for surgical treatment before treatment. And after treatment, HNC patient QOL increased along with oral function such as swallowing function between 1M to 3M. In this term, patients might relive a stress for surgical treatment and felt improve the physical function. This might be related the increasing of Global health status.

*Limitations*

This prospective cohort study included a small sample owing to its single-center design and loss to follow-up. Patient drop out during a prospective HNC study is not unusual (Rademaker et al., 2003; Shinn et al., 2013). In addition, patients were only followed for 3 months posttreatment. Postoperative dysfunction persists over 1 month and over 3-12 months after major surgery and radiation therapy, respectively (Chandu et al., 2006; Murphy et al., 2007). Furthermore, additional variables such as type, amount, and duration of medications (particularly pain medications) might have influenced the results. Thus, to better clarify the proposed patterns reported in the current study, future studies should incorporate larger samples, follow patients for a longer post-treatment duration, and consider additional variables that potentially influence the observed outcomes. In this study, we did not evaluate physical function such as walking speed, hand grip, and performance of activities of daily living. The correlation between the QOL of HNC patients who underwent surgical treatment and physical function should be investigated in future studies. In addition, it will be necessary that we consider classification by primary site (e.g., tongue, faucial arch, and pharynx) and identify the difference in treatment methods (surgery, radiation therapy, chemo therapy, and combined therapy).

In conclusion, muscle mass-related measurements, oral function measurements, and feeding function deteriorate significantly following surgical treatment for HNC and are not recovered completely at 3 months posttreatment. Furthermore, the different patterns of relationships between QOL measurements and oral functions or muscle mass-related measurements obtained at each assessment point indicate that different factors affect to the QOL in HNC patients who undergo surgical treatment. It is important to treat these patients in view of different factors affect to the QOL at each time point.

*Compliance with Ethics Standards*

The authors declare that they have no conflicts of interest.

• Research involving Human Participants and/or Animals: This study was approved by the Ethics Committee of Showa University School of Medicine (Approval no. 2355).

• Informed consent: Informed consent was obtained from all individual participants included in the study.
